# Investigating asphalt aggregate bonding degradation through aging and temperature effects using multiscale testing methods

**DOI:** 10.1038/s41598-025-26507-x

**Published:** 2025-11-27

**Authors:** Lemin Liu, Tao Liu, Xiaohua Fan, Hui Wei, Shengxu Wang, Liang Fan

**Affiliations:** 1 Shandong High-Speed Infrastructure Construction Co., Ltd., Jinan, 250098 China; 2 Shandong Transportation Research Institute, Jinan, 250100 China

**Keywords:** Asphalt-aggregate adhesion, Digital image analysis, Surface energy theory, Moisture damage resistance, Quantitative adhesion assessment, Engineering, Materials science

## Abstract

Moisture damage in asphalt mixtures is a critical durability concern influenced by interfacial adhesion between asphalt and aggregates. This study systematically evaluates adhesion performance using digital image analysis and surface energy theory across four asphalt types (OR50, OR70, OR90, ORSBS) and five aggregates (limestone, basalt, granite, diabase, diorite). Key findings reveal that limestone exhibits superior adhesion stability (100% adhesion rate under aging) due to chemical bonding, while acidic aggregates (e.g., granite) show significant sensitivity to moisture and aging. A novel quantitative method combining water immersion tests and pixel-based stripping area analysis demonstrated high correlation (R^2^ > 0.9) with surface energy-derived *ER* values, validating its reliability. Temperature and aging effects were further quantified, showing adhesion loss under thermal/oxidative aging, with SBS-modified asphalt outperforming base binders. These results provide a robust framework for material selection in pavement design, emphasizing aggregate alkalinity and polymer modification as key factors for moisture resistance.

## Introduction

The moisture damage of asphalt mixtures represents a complex phenomenon influenced by multiple factors including the properties of asphalt and aggregates, interfacial adhesion characteristics, void content in mineral aggregates, asphalt film thickness, and degree of asphalt saturation^1,2^. Among these factors, the interfacial bonding performance between asphalt and aggregates plays a decisive role in determining the moisture stability of asphalt pavement, as it directly governs the resistance against water-induced damage at the critical asphalt-aggregate interface^3–5^.

The adhesion between asphalt and aggregates is essentially a complex physicochemical phenomenon. Currently, four main theories are widely used to evaluate the adhesion mechanisms and methods between asphalt and aggregates: mechanical theory^6^, surface energy theory^7^, chemical reaction theory^8^, and molecular orientation theory^9^.

The boiling water test and water immersion test have become the most widely used methods for evaluating asphalt-aggregate adhesion due to their simple principles, straightforward operation, high repeatability, and easily interpretable results^10,11^. However, these methods present challenges in precisely controlling test conditions - for instance, maintaining the slight boiling state in the boiling water test is difficult, and different observers may assess the stripped surface differently. These significant human factors make it impossible to quantitatively evaluate the adhesion between asphalt and aggregates.

The net adsorption method employs ultraviolet spectrophotometry for measurement^12^. Its fundamental principle involves determining the concentration changes in the solution system before and after water addition. By measuring the corresponding absorbance variations, the initial adsorption capacity and net adsorption of asphalt onto aggregates can be calculated, ultimately yielding the asphalt-aggregate adhesion rate^13,14^. In the pre-hydration phase, aggregates adsorb asphalt molecules from the toluene solution, reducing the system concentration. Following water introduction, the system transforms into an asphalt-water-toluene ternary system, where water molecules displace asphalt from aggregate surfaces, consequently increasing the solution concentration^15^. Although the net adsorption method involves complex procedures, it offers high precision with minimal human interference, enabling quantitative assessment of asphalt-aggregate bonding performance.

The contact angle method (based on surface energy theory) provides a microscopic approach to analyze the interfacial bonding performance between asphalt and aggregates^16^. Essentially an energy conversion theory, surface energy theory postulates that the bonding strength at the asphalt-aggregate interface depends on the asphalt’s wettability on aggregate surfaces - complete wetting indicates strong bonding, while incomplete wetting suggests weak adhesion^17–20^. Wettability here refers to asphalt’s ability to coat aggregates, which is fundamentally governed by the physicochemical properties of both materials. In aqueous environments, water’s superior diffusivity enables it to rapidly coat aggregate surfaces, displacing the asphalt film through competitive adsorption and consequently degrading the interfacial bonding performance^21–24^.

Several researchers have advanced this methodology: Little^25^, Hefer^6^, and Bhasin^26,27^ employed surface free energy theory to analyze moisture susceptibility in asphalt mixtures; Little and Bhasin^28^ utilized the Wilhelmy plate method to measure contact angles between solids and liquids for surface energy calculations.

While boiling water and immersion tests are widely used, their subjective interpretation and inability to quantify adhesion limit reliability. Surface energy theory, though precise, requires complex instrumentation unsuitable for routine practice. This study bridges the gap by proposing a hybrid approach: digital image analysis for quantitative stripping area measurement, validated against surface energy theory. We further systematically evaluate temperature and aging effects across diverse asphalt-aggregate combinations, providing actionable insights for pavement durability.

To facilitate convenient and quantitative characterization of adhesion between different asphalt-aggregate combinations, this study employed four asphalt types and five aggregate varieties. The digital image processing technique was utilized to quantify the stripping rate of asphalt from aggregate surfaces after water immersion. The results were subsequently correlated with surface energy measurements to validate the reliability of this methodology. Furthermore, the approach was systematically verified for its applicability across varying temperature conditions and aging states.

This study proposes a novel hybrid approach combining digital image analysis and surface energy theory to quantitatively assess asphalt-aggregate adhesion. By using digital image processing to measure stripping areas, it addresses the limitations of traditional tests. The approach is further validated across different temperatures and aging conditions, offering a more reliable and practical method for evaluating the moisture stability of asphalt pavements.

## Materials and methods

### Raw materials

The study selected a total of four different types of asphalt (OR50, OR70, OR90, SBS) and five types of aggregates (limestone, basalt, granite, diabase, diorite) for adhesion analysis. The basic properties of the four asphalt types are shown in Table 1.

**Table 1:** Basic properties of four kinds of asphalt.

Properties	OR50	OR70	OR90	ORSBS
Softening point/°C	51.0	48.5	45.0	76.5
Penetration (25 °C)/0.1 mm	47	67	84	46
Ductility (10 °C)/cm	16	37	> 100	48.7
Dynamic viscosity (135 °C)/ Pa⋅s	0.66	0.56	0.48	1.87

The mineral composition of the five types of aggregates is shown in Table 2.

**Table 2:** Mineral composition of aggregates (%).

Aggregate type	SiO_2_	CaO	MgO	Al_2_O_3_	Fe_2_O_3_
Limestone	15.5	27.7	18.78	2.54	1.59
Basalt	53.99	7.02	4.9	15.5	8.88
Granite	72.77	1.43	0.22	14.8	1.16
Diabase	49.95	8.5	5.76	13.84	8.87
Diorite	60.24	5.83	3.86	18.45	4.26

To investigate the changes in adhesion performance between asphalt and aggregates after aging, both short-term and long-term aging treatments were conducted on the asphalt binders.

Short-Term Aging: The rolling thin film oven (RTFOT) method was employed for short-term aging. A 35 g asphalt sample was placed in a glass container and aged in the RTFOT at 163 °C. The oven rotated at 15 r/min with a heated air flow rate of 4000 mL/min for a duration of 85 min.

Long-Term Aging: The pressure aging vessel (PAV) was used for long-term aging. Approximately 50 g of the short-term aged asphalt was transferred into a PAV pan and aged at 105 °C under compressed air at 2.1 MPa ± 0.1 MPa for 20 h.

The aged samples were designated as follows:

Short-term aged samples: OR50 → TR50, OR70 → TR70, OR90 → TR90, ORSBS → TRSBS.

Long-term aged samples: OR50 → PR50, OR70 → PR70, OR90 → PR90, ORSBS → PRSBSS.

### Research methodology

#### Asphalt four-component analysis

Based on the *Test Methods for Bitumen and Bituminous Mixtures in Highway Engineering* (JTG E20-2011)^29^ four-component separation standard, TLRC achieves component separation through differential migration distances in specific solvent systems, followed by chromatographic scanning for quantitative analysis of component contents. Based on the test results, the asphalt was fractionated into four components: saturates (*S*), aromatics (*Ar*), resins (*R*), and asphaltenes (*At*).

1. Preparation: Activate the chromatographic rods. Dissolve the sample in toluene to prepare a 20 mg/mL asphalt solution.
2. Sample Application: Spot the sample at the origin point using a micro-syringe.
3. Development Process: Immerse the rod holder in Developer #1 until reaching target migration height. Dry for 5 min using air-blowing device. Condition in humidity chamber for 10 min. Repeat the process with Developer #2 (including drying and conditioning steps). Final development with Developer #3, followed by 10-minute drying.
4. Scanning: Place dried rods in chromatographic analysis workstation. Perform scanning to obtain component separation profiles.
5. Data Analysis: Analyze chromatograms to determine separated component contents.
6. Four-component analysis was performed on the asphalt samples both before and after aging.

#### Water immersion test

The water immersion test was conducted in accordance with JTG E20-2011. The detailed procedure is as follows:

1. Preparation of Asphalt Binder: Heat the asphalt sample to the specified mixing temperature (180 °C).
2. Aggregate Preparation: Sieve the aggregates to obtain particles sized between 9.5 mm and 13.2 mm. Wash 200 g of the selected aggregates (preferably flat and regular-shaped) with clean water, dry them in a desiccator, and then divide them using the quartering method to obtain a 100 g sample. Place the 100 g sample in a mixing container and heat it in an oven at 185 °C for 1 h.
3. Asphalt-Aggregate Mixing: Weigh 5.5 ± 0.2 g of asphalt (accurate to 0.1 g) per 100 g of aggregates. Pour the weighed asphalt into the mixing container with the heated aggregates. Return the container to the oven and heat for an additional 15 min.
4. Mixing and Cooling: Remove the container from the oven and stir thoroughly with a glass rod for 1–1.5 min until the asphalt is uniformly coated on the aggregate surfaces. Transfer the mixture to a glass plate using a small spatula and allow it to cool at room temperature for 1 h before the water immersion test.
5. Water Immersion Test: Submerge the prepared sample in a constant-temperature water bath maintained at 80 ± 1 °C. Immediately remove any loosened or floating asphalt film from the water surface.
6. Evaluation of Adhesion: After 30 min, carefully remove the glass plate from the water bath and immerse it in pre-prepared cold water.

Observe the extent of asphalt film stripping, estimate the stripping ratio, and classify the adhesion performance according to the specified rating scale.

#### Digital image analysis of adhesive failure

The adhesion performance was evaluated using Photoshop-based pixel analysis to determine the stripped or adhered asphalt area on aggregate surfaces after water immersion. The procedure is as follows:

1. Sample Preparation:

Select five flat-surfaced aggregates for water immersion testing.

2. Image Acquisition:

Photograph the post-immersion aggregates under uniform, high-intensity lighting using a macro lens to ensure consistent clarity.

3. Image Processing and Thresholding:

Since the asphalt regions appeared as black areas in the images, we applied a color-based selection strategy rather than grayscale conversion. Specifically, the Color Range / Select Color tool in Photoshop was used to isolate the black asphalt regions.

(1) Thresholding was performed in the RGB channels, with the following settings: Black asphalt regions were identified by selecting pixels within the low RGB value range (R: 0–50, G: 0–50, B: 0–50).

The contrast between the dark asphalt and the lighter-colored aggregates was enhanced by adjusting the selection fuzziness between 25 and 35 to ensure precise isolation of asphalt pixels without including aggregate edges.

(2) Manual fine-tuning was performed when necessary to ensure that all asphalt areas were accurately captured, especially at aggregate boundaries.

4. Boundary Handling and Error Minimization:

To address irregular aggregate edges, a contour smoothing step was applied after pixel selection to reduce jagged boundaries. Additionally, a light Gaussian blur (radius = 2 px) was applied to reduce pixel-level noise before quantification.

5. Calculation: Compute the following ratios.

Stripping ratio (%) = (Stripped area pixels / Total surface pixels) × 100.

Adhesion ratio (%) = (Adhered asphalt pixels / Total surface pixels) × 100.

**Fig. 1:**
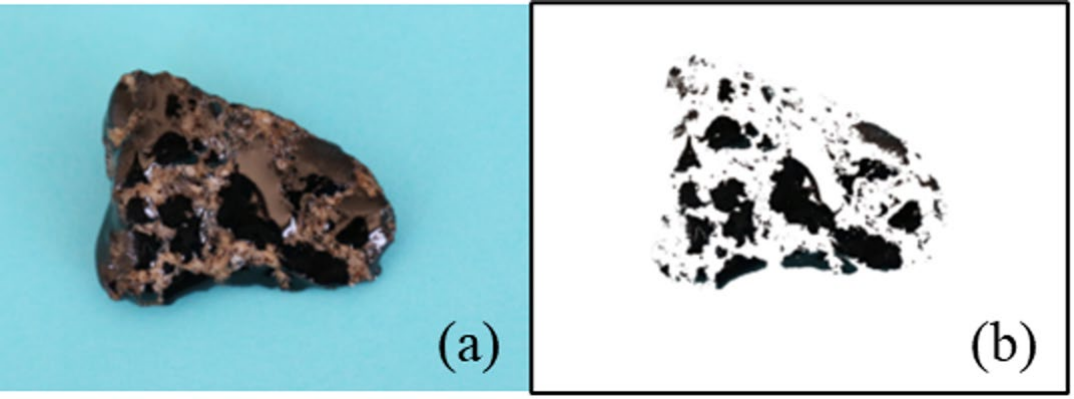
Image processing effect. (**a**) Original image; (**b**) processed image (asphalt isolated).

The image processing effect is shown in Fig. 1.

#### Surface energy measurements

To validate the rationality and stability of the quantitative evaluation method, the quantitative test results were compared and analyzed with the surface energy-based moisture susceptibility evaluation index ***ER*** (Eq. 1)^30^.

1 $$\:ER=\left|\frac{{W}_{as}}{{W}_{aws}}\right|$$

where: *ER*: Moisture susceptibility evaluation index; *W*_*as*_: Work of adhesion (mJ/m^2^); *W*_*aws*_: Work of debonding (mJ/m^2^).

The calculation formulas for *W*_*as*_ and *W*_*aws*_ are given in Eqs. (2) and (3), respectively.

2 $$\:{W}_{as}={\gamma\:}_{a}\left(1+\text{cos}\theta\:\right)$$

3 $$\:{W}_{asw}={\gamma\:}_{a}\left(1+\text{cos}\theta\:\right)-{\gamma\:}_{w}\text{cos}{\theta\:}_{1}-{\gamma\:}_{w}\text{cos}{\theta\:}_{2}$$

In the equation, $\:{\gamma\:}_{a}$ represents the surface energy of asphalt (mJ/m^2^), $\:{\gamma\:}_{w}$ denotes the surface energy of water (mJ/m^2^), *θ* is the contact angle between asphalt and aggregate (°), *θ*_*1*_ indicates the contact angle between water and asphalt (°), and *θ*_*2*_ stands for the contact angle between water and aggregate (°).

**Fig. 2:**
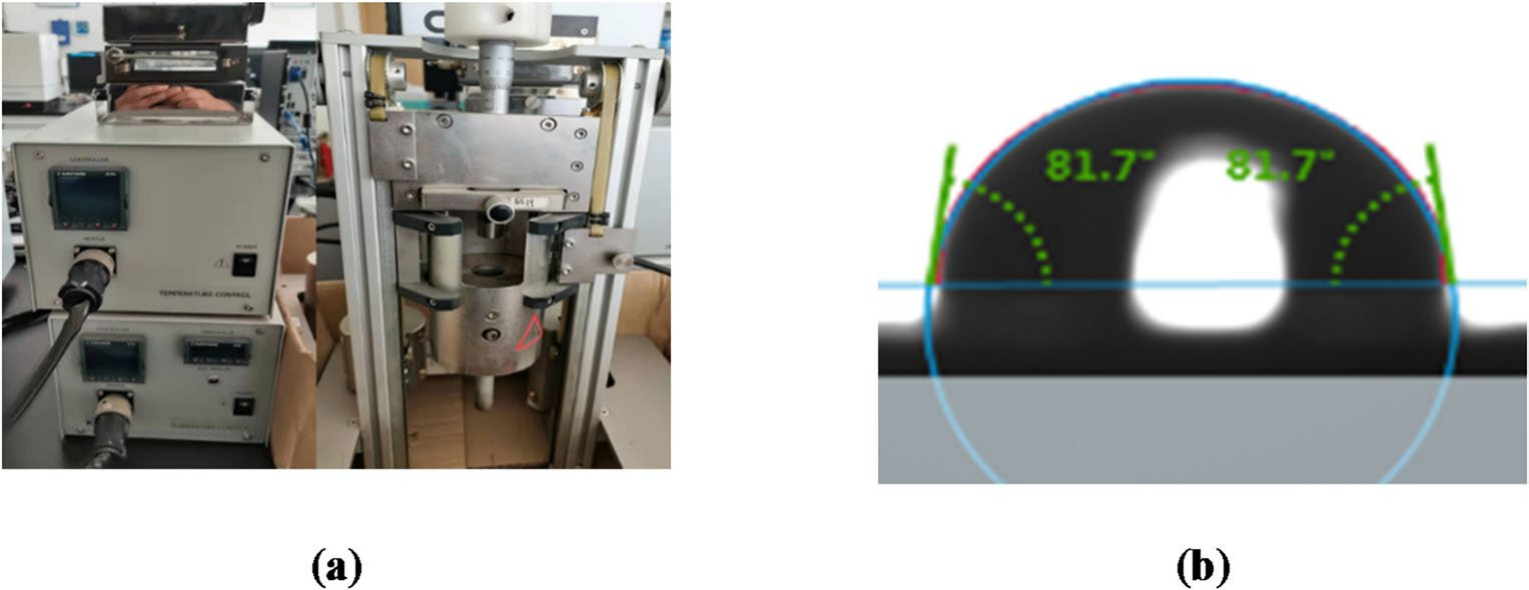
The contact angle testing equipment and its schematic diagram: (**a**) contact angle testing equipment (with high-temperature goniometer); (**b**) schematic diagram of sessile drop method.

The contact angle measuring instrument (KRUSS-DSA100) was selected as the testing equipment, employing the sessile drop method to measure the contact angles between water/bitumen and aggregates. The contact angle testing equipment and its schematic diagram are illustrated in Fig. 2.

The dynamic contact angle was quantified at 155 ± 1 °C using a high-temperature goniometer, replicating the actual asphalt-aggregate interfacial conditions during hot mixing. The contact angle changes dynamically over time. In this study, the contact angle at 10 s was chosen to calculate the surface energy, in order to avoid the influence of instantaneous fluctuations caused by the droplet’s contact with the surface.

## Results

### Quantitative evaluation of asphalt-aggregate adhesion

The water immersion tests were conducted on all five aggregate types, with the quantitative results presented in Table 3.

**Table 3:** Adhesion grades between different aggregates and asphalt.

Aggregate type	OR50	OR70	OR90	ORSBS
Limestone	5*	5	5	5
Basalt	3	2 ~ 3	4	5
Granite	2 ~ 3	2 ~ 3	2 ~ 3	4
Diabase	5	4	4	5
Diorite	4	3	3	5

*A higher numerical value indicates better adhesion performance.

As shown in Table 3, the visual adhesion grading obtained through the water immersion test exhibits relatively low precision, providing only approximate ranges. Moreover, this method is highly susceptible to subjective human judgment, resulting in limited accuracy and an inability to quantitatively evaluate the interfacial bonding performance between asphalt and aggregates.

**Fig. 3:**
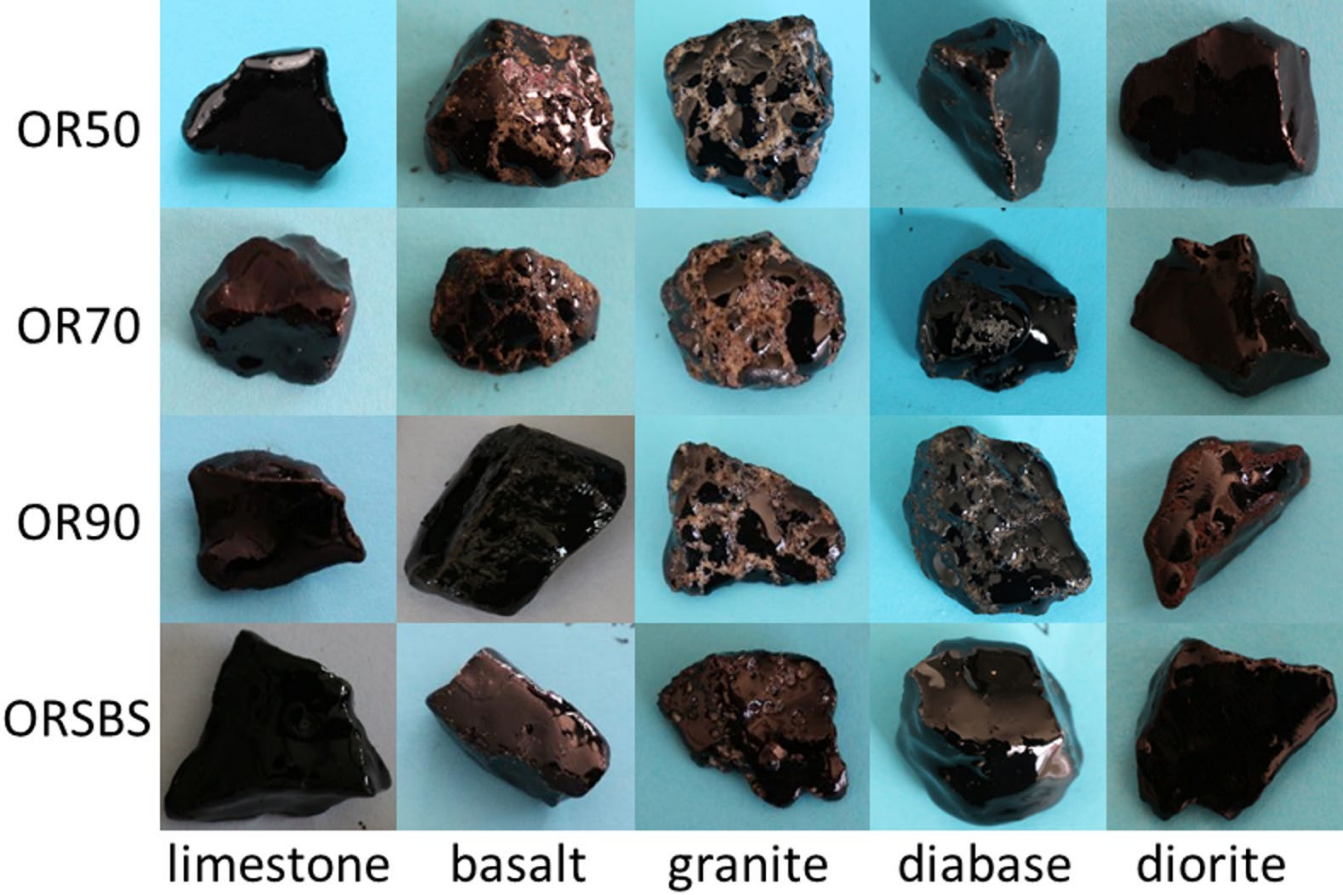
Test results of asphalt-aggregate samples after water immersion.

To address these limitations, the post-immersion aggregates were photographed, and the images were processed using Photoshop pixel analysis. This approach calculates the adhesion ratio, enabling a quantitative assessment of the asphalt-aggregate bonding characteristics. Representative images of the immersed aggregates are presented in Fig. 3.

**Fig. 4:**
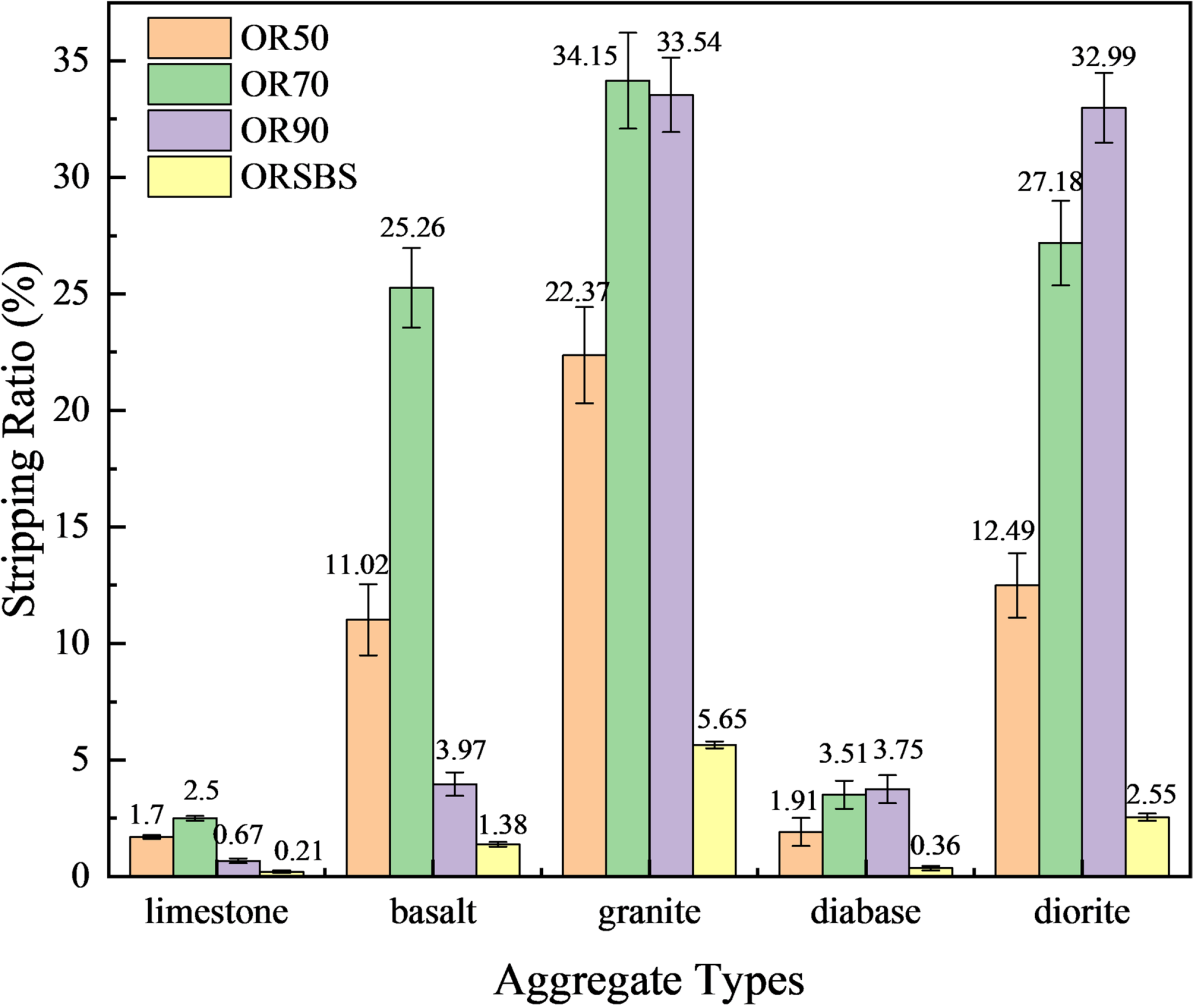
Stripping rates of different asphalt-aggregate combinations.

The acquired images were analyzed using the Photoshop pixel area method, where the stripping ratio was calculated according to the methodology described in Sect. 2.1.2. The average results of ten experiments conducted under each experimental condition are recorded in Fig. 4.

Figure 4 demonstrates a clear adhesion performance ranking between the same asphalt grade and different aggregates, following the order of limestone > diabase > basalt > diorite > granite, which directly corresponds to their alkalinity levels from highest to lowest. The superior adhesion of limestone, composed primarily of CaCO_3_, stems from its strong chemical bonding with asphalt’s acidic components, showing minimal variation across test points due to this effective chemisorption. In contrast, diabase and basalt, containing approximately 50% SiO_2_ and classified as neutral aggregates, exhibit 20–30% lower adhesion than limestone because of their limited chemical reactivity. Granite, with about 70% SiO_2_ content and acidic nature, displays the poorest adhesion performance as it lacks chemical affinity with asphalt and relies solely on mechanical interlocking. When examining different asphalt types with the same aggregate, the results reveal that the penetration grade of base asphalt does not significantly influence adhesion performance.

The successful application of digital image analysis through Photoshop pixel quantification objectively verifies these interfacial bonding characteristics, confirming the method’s sensitivity to aggregate mineralogy, measurement repeatability, and superior reliability compared to traditional visual assessment techniques. These findings highlight the critical role of aggregate chemistry in pavement durability while demonstrating the effectiveness of quantitative image analysis for evaluating asphalt-aggregate compatibility.

### Adhesion test results based on surface energy

#### Test results of contact angle between asphalt and aggregates

**Fig. 5:**
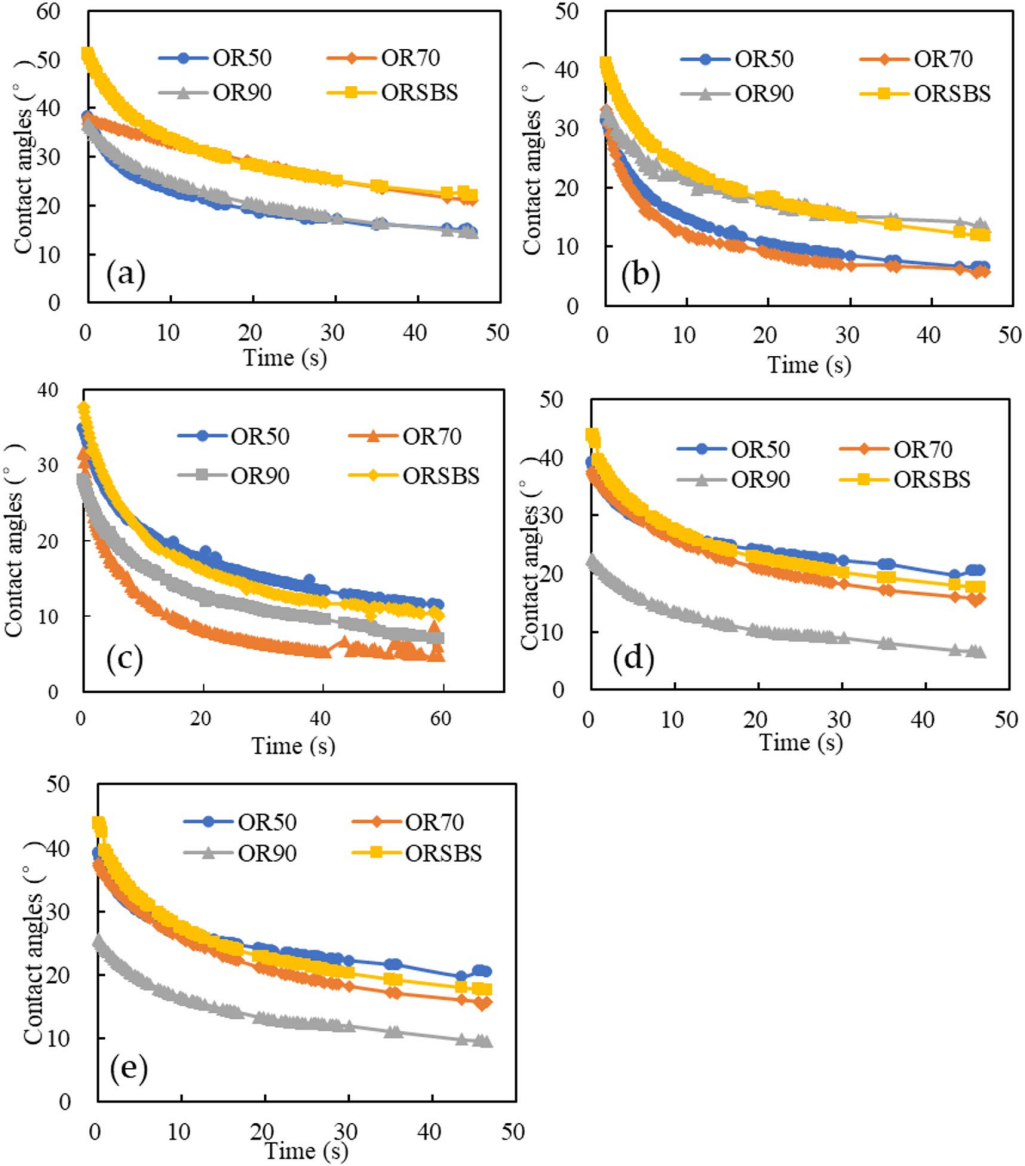
Temporal evolution of contact angles for different asphalt-aggregate combinations (**a**) limestone; (**b**) basalt; (**c**) granite; (**d**) diabase; (**e**) diorite.

The spreading kinetics of distinct asphalt binders on aggregate surfaces exhibited significant variations, necessitating dynamic contact angle monitoring until stabilization. As evidenced in Fig. 5, the equilibrium contact angles were attained through distinct temporal patterns.

As observed in Fig. 4, the contact angles between all aggregates and asphalt gradually decreased over time before slowly stabilizing. The asymptotic contact angle behavior reflects thermodynamic equilibrium attainment, where: Limestone’s higher final θ (20°-30°) indicates weaker wettability but stronger chemisorption. Granite’s lower θ (5°-15°) manifests better wetting but poorer chemical bonding. This aligns with the stripping ratio ranking in Sect. 3.1.

#### The surface energy-based moisture susceptibility evaluation

**Fig. 6:**
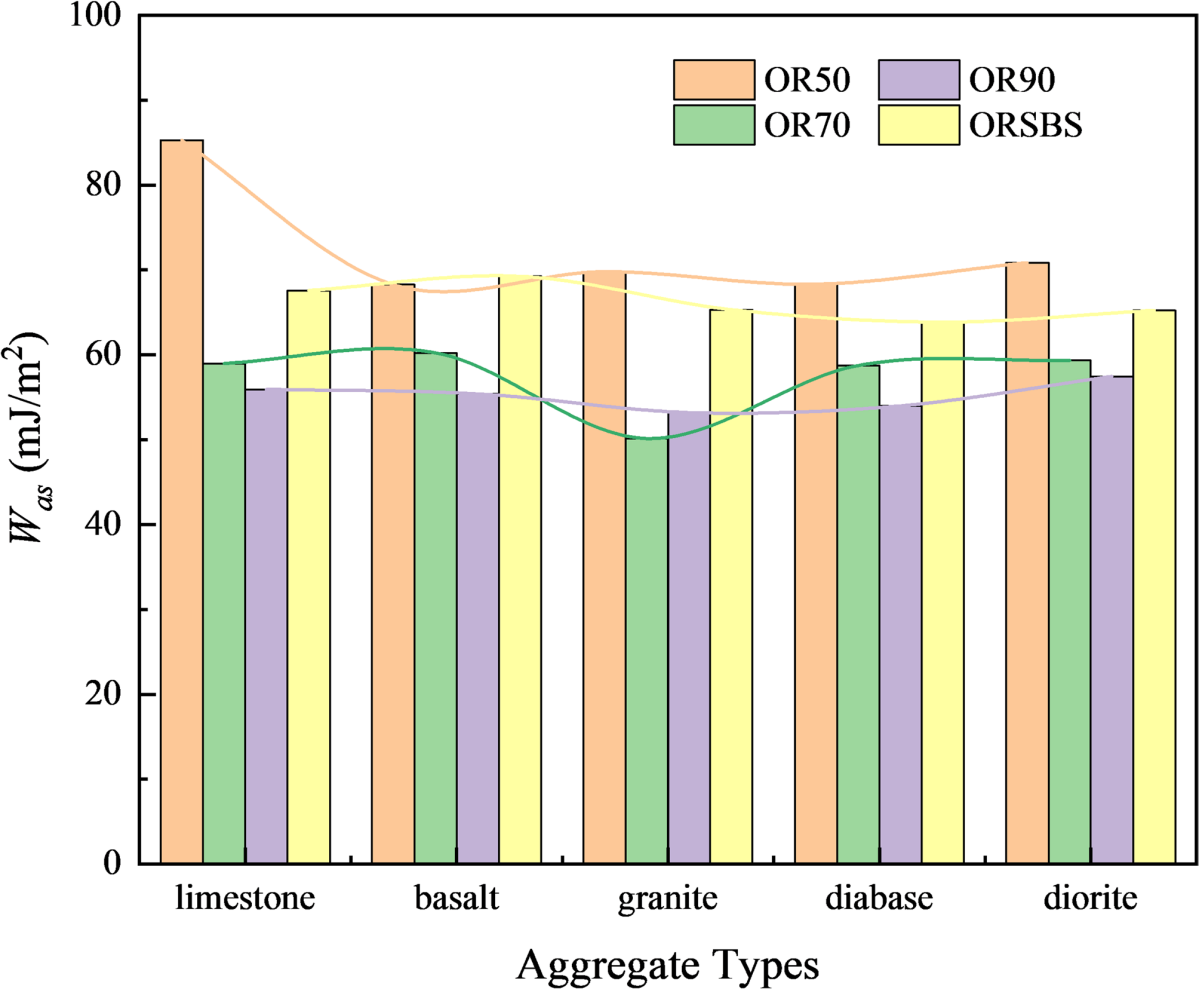
Work of adhesion (*W*_*as*_) under dry conditions.

**Fig. 7:**
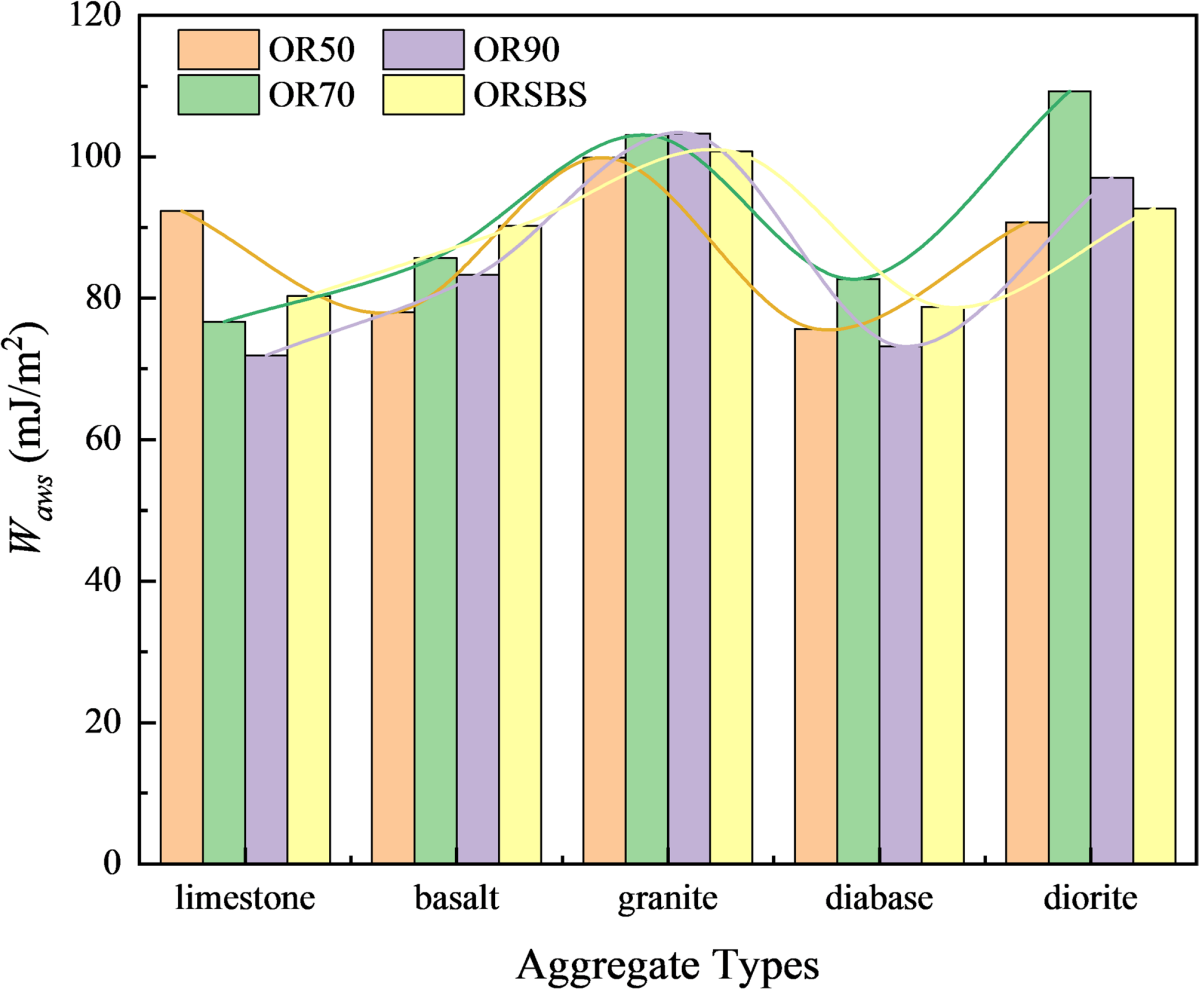
Work of debonding (*W*_*aws*_) after water exposure.

Based on the results in Fig. 5, the work of adhesion (*W*_*as*_) and the work of debonding (*W*_*aws*_) were calculated using Eqs. 2 and 3, respectively. The computational results are presented in Figs. 6 and 7.

As shown in Fig. 6, the work of adhesion (*W*_*as*_) varies significantly between different asphalt binders and the same aggregate type. Taking OR70 asphalt as an example, the adhesion performance follows the order: basalt > limestone > diabase > diorite > granite, which aligns with conventional understanding that alkaline aggregates exhibit better adhesion with asphalt. However, OR50 asphalt shows a completely different trend: limestone > diorite > granite > basalt > diabase.

This discrepancy suggests that using *W*_*as*_ alone to evaluate asphalt-aggregate adhesion has certain limitations. While OR70’s results conform to the established rule (higher adhesion with alkaline aggregates), OR50’s reverse trend indicates other factors (e.g., asphalt chemical composition, surface energy characteristics) may dominate the interfacial bonding behavior in some cases.

The results presented in Fig. 7 demonstrate a consistent hierarchy in the work of debonding (*W*_*aws*_) across different asphalt-aggregate combinations, with limestone exhibiting the strongest moisture resistance, followed by diabase, basalt, diorite, and granite, confirming that alkaline aggregates maintain superior adhesion with asphalt in the presence of water while acidic aggregates perform poorest. This thermodynamic parameter *W*_*aws*_, which specifically quantifies water’s disruptive effect on the asphalt-aggregate interface, proves more reliable than dry adhesion measurements for evaluating moisture susceptibility, as evidenced by its strong correlation with both laboratory performance and real-world pavement behavior where limestone-based mixtures consistently outperform granite counterparts in wet conditions. The robust agreement between these surface energy measurements and practical engineering experience validates *W*_*aws*_ as a fundamentally sound indicator for predicting water damage potential in asphalt mixtures.

The energy ratio (*ER*), as a comprehensive moisture susceptibility indicator, was derived from the interfacial energy parameters (*W*_*as*_ and *W*_*aws*_) in Figs. 5 and 6 through Eq. 1. Table 4 systematically presents the calculated *ER* values.

**Table 4:** Energy ratio (*ER*) values for different asphalt-aggregate combinations.

Aggregate type	OR50	OR70	OR90	ORSBS
Limestone	0.924	0.769	0.776	0.841
Basalt	0.876	0.703	0.665	0.767
Granite	0.698	0.486	0.515	0.648
Diabase	0.90	0.701	0.737	0.810
Diorite	0.781	0.543	0.592	0.703

From the data in Table 4, it can be observed that basic aggregates generally maintain higher *ER* values, while acidic aggregates show lower *ER* values. The alkalinity/acidity of aggregates remains a key factor influencing their resistance to moisture damage.

### Consistency analysis between quantitative analysis methods and ER value results

**Fig. 8:**
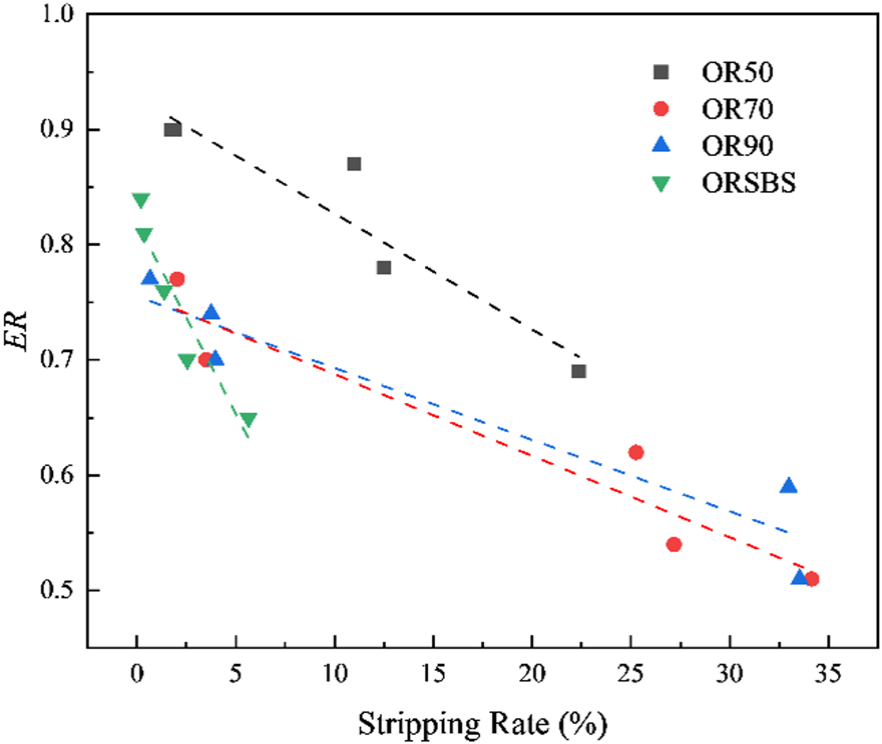
Correlation analysis between stripping rate and *ER*.

To validate the applicability and consistency of the quantitative results obtained from the water-immersion-based digital image analysis method, a correlation analysis was conducted between the stripping rate calculated in Sect. 3.1 and the moisture damage evaluation index (ER) from Sect. 3.2.2. The results are shown in Fig. 8.

The curve-fitting equations and variances for different asphalt binders in Fig. 8 are recorded in Table 5.

**Table 5:** Linear fitting equations and variances for different asphalt binders.

Asphalt binders	Fitting equations	*R* ^2^
OR50	y = -0.0101x + 0.9276	0.891
OR70	y = -0.0071x + 0.7581	0.912
OR90	y = -0.00621x + 0.7550	0.910
ORSBS	y = -0.0331x + 0.8191	0.898

The statistical results in the table demonstrate a strong correlation between the stripping rate obtained through digital image analysis and the surface energy-based moisture damage evaluation index (*ER*). Notably, both OR70 and OR90 exhibit correlation coefficients exceeding 0.9. This indicates that the combined approach of water immersion testing and image processing technology provides an efficient method for evaluating the adhesion between asphalt and aggregates, with results demonstrating both stability and consistency.

### Effect of temperature on adhesion performance

**Fig. 9:**
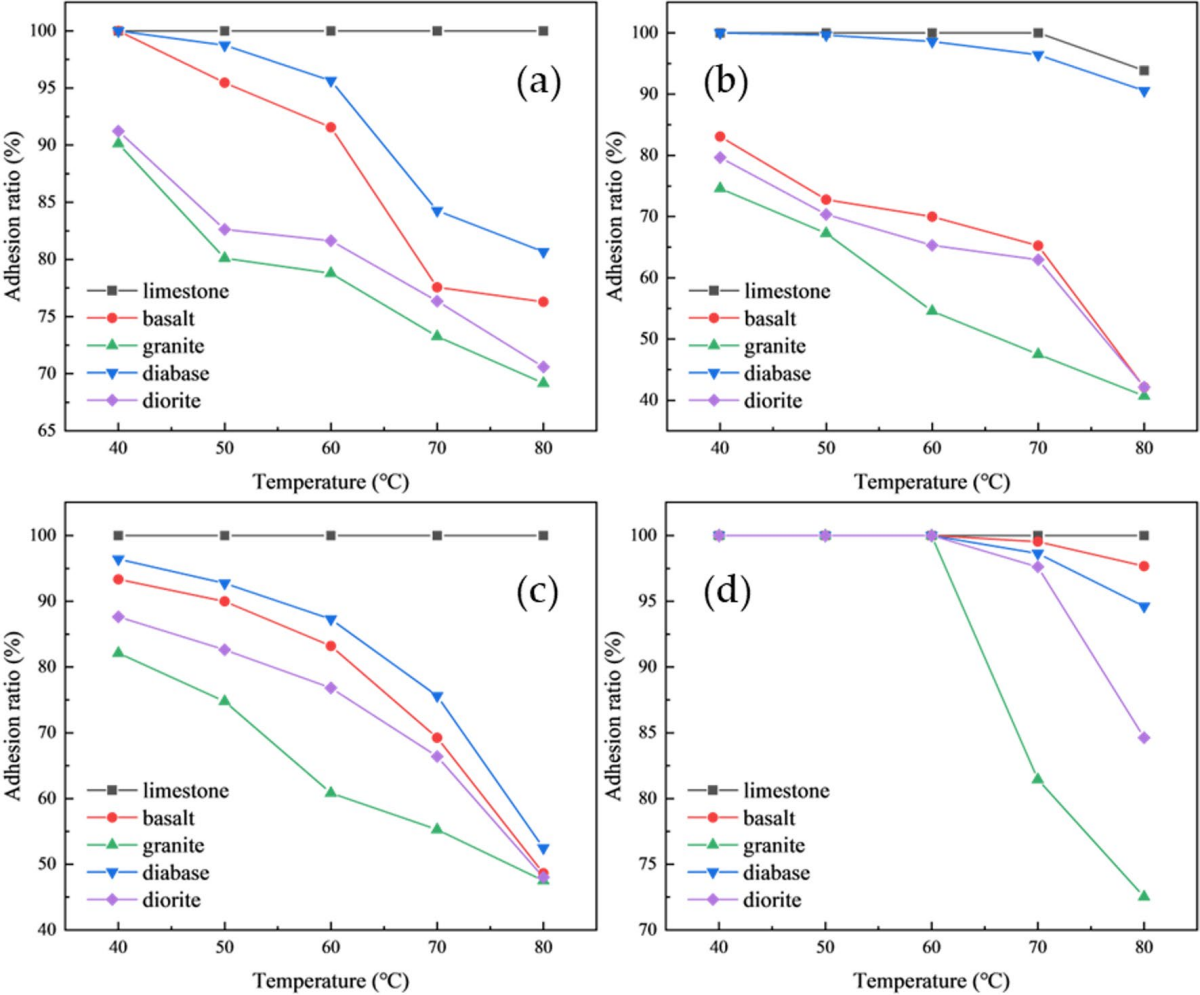
Quantitative analysis of temperature-dependent adhesion performance using digital image processing (**a**) OR50; (**b**) OR70; (**c**) OR90; (**d**) ORSBS.

This section investigates the water immersion test at bath temperatures of 40 °C, 50 °C, 60 °C, 70 °C, and 80 °C. The stripping area at each temperature was quantified using pixel area analysis, with the resulting adhesion rates shown in Fig. 9.

Figures 9 shows the adhesion rates between various asphalt binders and aggregate types under different temperature conditions. The results demonstrate that the adhesion performance generally follows the order of limestone > diabase > basalt > diorite > granite. Limestone exhibits exceptional temperature stability, maintaining nearly 100% adhesion across all tested temperatures, while diabase, basalt, diorite and granite show significant temperature-dependent variations in adhesion performance.

The critical temperature at which stripping occurs differs among various asphalt-aggregate combinations. A clear trend emerges where adhesion rates progressively decrease with rising temperature. This phenomenon can be attributed to thermal expansion increasing intermolecular distances and consequently weakening interfacial forces. At elevated temperatures, the enhanced polarity of aggregates strengthens their water adsorption capacity, leading to competitive displacement of asphalt films by water molecules. As temperature increases, this water-asphalt competition results in gradual film detachment from aggregate surfaces, manifested as reduced adhesion rates. Ultimately, these processes degrade the interfacial bonding performance between asphalt and aggregates, thereby diminishing the mixture’s resistance to moisture damage.

The comprehensive data presented in these figures provides valuable insights into the temperature sensitivity of different asphalt-aggregate systems, particularly highlighting limestone’s superior performance stability compared to other common aggregate types under thermal variations. These findings have important implications for pavement material selection in different climatic conditions.

The temperature at which stripping occurs varies for different asphalt-aggregate combinations. As shown in Fig. 9, limestone demonstrates superior adhesion with asphalt, requiring higher temperatures to induce stripping, whereas granite and basalt exhibit poorer adhesion performance with stripping occurring at relatively lower temperatures. Therefore, when employing the water immersion method to evaluate asphalt-aggregate adhesion, it is recommended to use different water bath temperatures for different aggregate types. For instance, a water immersion temperature of 80 °C may be considered for limestone, while 40 °C would be more appropriate for basalt and granite.

### Effect of aging on adhesion performance

#### Changes in asphalt components before and after aging

**Fig. 10:**
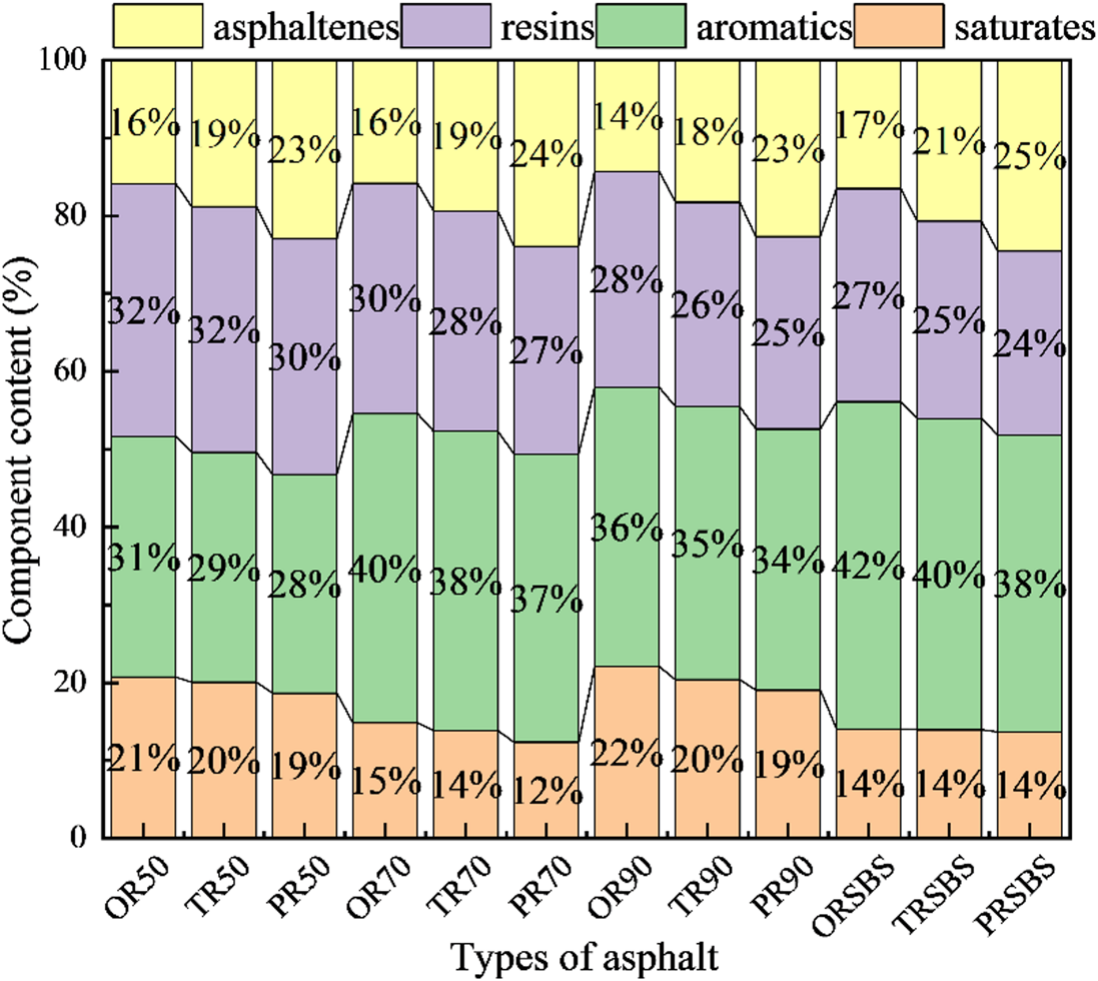
Changes in four-component composition of aged asphalt.

The changes in component contents of various asphalts after aging are presented in Fig. 10, which clearly shows a progressive decrease in saturates, aromatics and resins contents coupled with a corresponding increase in asphaltenes content as the aging process intensifies. Among these components, saturates demonstrate the highest resistance to aging effects, exhibiting minimal content variations of less than 1% under different aging conditions. In contrast, asphaltenes display the most pronounced sensitivity to aging, with short-term aging resulting in an increase of at least 3% and long-term aging causing a more substantial growth ranging between 7% and 9%. These quantitative changes in component distribution provide direct evidence of the chemical transformations occurring during asphalt aging, particularly the oxidative conversion of lighter fractions into heavier asphaltenes compounds.

#### Changes in adhesion between asphalt and aggregates

**Fig. 11:**
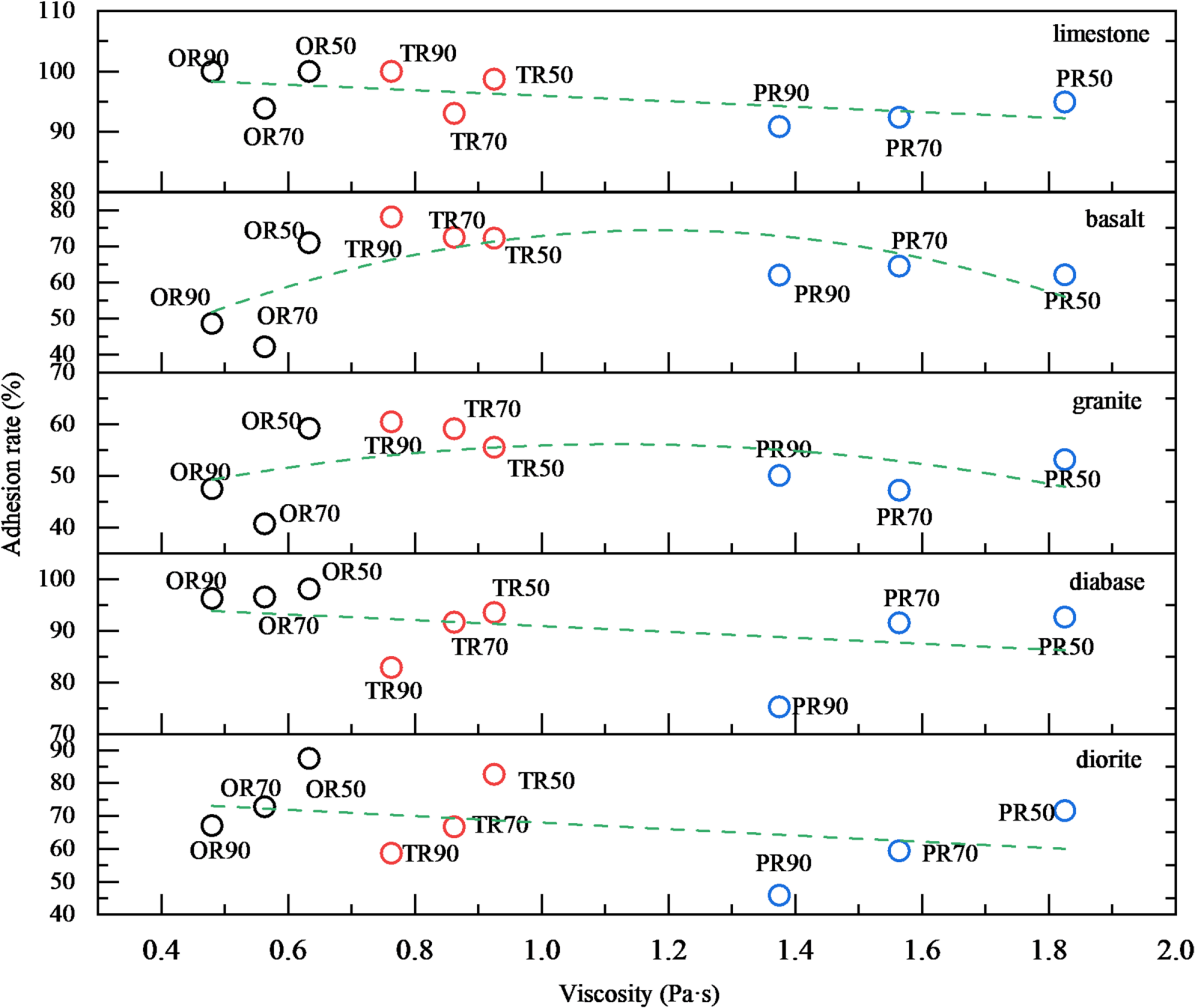
Effect of base asphalt aging on adhesion rate.

The stripping areas at different aging levels were quantified using digital image analysis to determine adhesion rates, as shown in Fig. 11. Since the viscosity of asphalt undergoes significant changes after aging and is generally considered to affect the adhesion rate, the dynamic viscosity at 135 °C was selected as the abscissa variable to investigate their correlation in this study.

Figure 11 present the adhesion rates between base asphalt and limestone, diabase, and diorite aggregates at different aging levels. The results demonstrate a gradual but moderate decrease in asphalt-aggregate adhesion as the base asphalt undergoes aging.

The interfacial adhesion between asphalt and aggregates involves complex physicochemical interactions. While aging enhances mechanical interlocking forces, it simultaneously alters the asphalt’s composition and structure, disrupting its original equilibrium. These changes reduce the asphalt’s surface activity and wetting capability, ultimately leading to diminished adhesion performance with progressive aging.

The adhesion rates between base asphalt and basalt/granite aggregates under various aging conditions, revealing a distinctive pattern where adhesion initially increases before declining with prolonged aging. This behavior contrasts with other aggregate types and can be attributed to the unique physical adsorption mechanism governing the asphalt-basalt/granite interface. As the asphalt undergoes aging, its compositional and structural changes disrupt the original colloidal equilibrium, while simultaneously enhancing mechanical interlocking forces through increased asphaltene content (rising more than 3%). When exposed to moisture, water molecules initially displace the asphalt film, but the aged asphalt’s improved mechanical anchoring capability facilitates re-adsorption onto the aggregate surface, creating additional binding sites and temporarily boosting adhesion performance. However, with continued aging, the progressive deterioration of asphalt’s wetting properties and surface activity ultimately leads to the observed decline in adhesion, demonstrating the complex interplay between physical interlocking and chemical bonding in these systems.

**Fig. 12:**
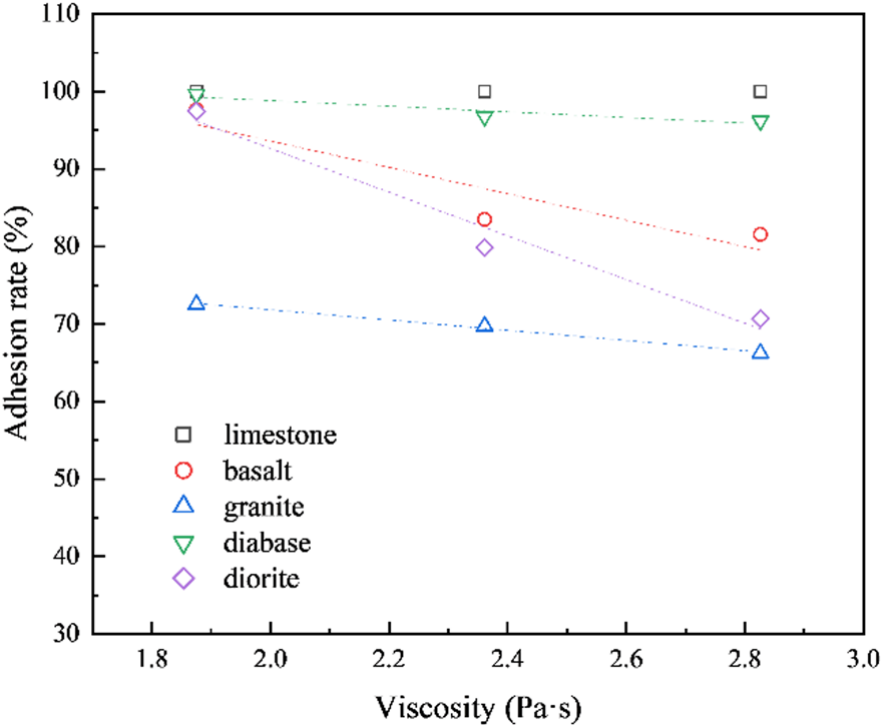
Adhesion rate of SBS-modified asphalt at different aging levels.

Figure 12 specifically presents the relationship between viscosity and adhesion rate changes in SBS-modified asphalt after aging. Due to SBS’s significantly higher viscosity compared to base asphalt (typically 2–3 times greater at 135 °C), its aging characteristics exhibit distinct patterns from unmodified binders. The plotted data reveals how the substantial viscosity increase during SBS aging - from approximately 1.8 Pa s (unaged) to over 2.8 Pa s (after PAV aging)—correlates with corresponding adhesion rate variations.

As shown in the Fig. 12, the SBS-modified asphalt maintains a constant 100% adhesion rate with limestone aggregates regardless of aging severity, indicating superior interfacial compatibility. This exceptional performance stems from the mechanical interlocking forces between aged SBS asphalt and limestone surpassing the chemical bonding effects, thereby preserving adhesion integrity. In contrast, the adhesion rates progressively decline for diabase, basalt, diorite, and granite aggregates with extended aging. This deterioration results from two synergistic mechanisms: firstly, aging degrades the polymer modifier’s effectiveness, causing structural and compositional changes that reduce crosslinking between asphalt and SBS modifiers, consequently weakening cohesive strength; secondly, while aging enhances mechanical adhesion, it simultaneously diminishes asphalt’s surface activity and wetting capability, ultimately impairing overall adhesion performance with these aggregate types.

## Conclusions

In summary, this study provides a comprehensive evaluation of asphalt-aggregate adhesion mechanisms through advanced quantitative methods, revealing critical insights into moisture damage resistance under varying environmental and aging conditions. The main findings and recommendations are as follows:

Limestone, with its CaCO₃-rich surface, demonstrates stable adhesion (100% under aging), while granite, due to its high SiO_2_ content, shows poor performance mainly due to reliance on mechanical interlocking. SBS-modified asphalt improves adhesion with granite, highlighting the importance of polymer modification in enhancing interfacial bonding.

Aging reduces adhesion by approximately 10%, primarily due to increased asphaltenes and resin depletion, which degrade wettability. SBS modification mitigates these effects, improving performance and maintaining adhesion strength, especially with granite aggregates.

Adhesion decreases with temperature, with granite failing at 40 °C and limestone at 80 °C, suggesting that testing conditions should be tailored to specific aggregates. Alkaline aggregates and SBS-modified asphalt are recommended for areas with high moisture exposure.

Strong correlation (R^2^ > 0.9) is observed between stripping areas and surface energy values, providing a reliable alternative to subjective visual assessments. This method should be incorporated into quality control for better accuracy in measuring asphalt-aggregate adhesion.

**Author contributions**.

## Data Availability

Data is contained within the article.
